# Analysing outbreak signals, 2013–2024: The amsterdam UMC centre for Tropical Medicine and Travel Medicine Epi Alert programme – an observational study

**DOI:** 10.1016/j.nmni.2026.101756

**Published:** 2026-05-04

**Authors:** Jelmer van Os, Hanna K. de Jong, Galadriel Pellejero-Sagastizabal, José Ramón Paño-Pardo, F-Xavier Lescure, Thomas Hanscheid, Martin P. Grobusch, Abraham Goorhuis

**Affiliations:** a Centre for Tropical Medicine and Travel Medicine, Department of Infectious Diseases, Amsterdam UMC Location University of Amsterdam, Meibergdreef 9, 1105, AZ Amsterdam, the Netherlands; b Servicio de Enfermedades Infecciosas, Division of Infectious Diseases, Hospital Clínico Universitario Universidad de Zaragoza, Instituto de Investigación Sanitaria Aragón (IIS Aragón), CIBERINFEC, 50009, Zaragoza, Spain; c Emerging Infections Subcommittee, European Society of Clinical Microbiology and Infectious Disease, Switzerland; d Infectious and Tropical Diseases Department, APHP, Bichat Hospital and Université Paris Cité, Inserm, IAME, F-75018, Paris, France; e Universidade de Lisboa, Faculdade de Medicina, Instituto de Microbiologia, Lisboa, Portugal; f Masanga Medical Research Unit (MMRU), Masanga, Sierra Leone; g Institute of Tropical Medicine, German Centre for Infection Research (DZIF), University of Tübingen, Tübingen, Germany; h Centre de Recherches Médicales en Lambaréné (CERMEL), Lambaréné, Gabon; i Institute of Infectious Diseases and Molecular Medicine (IDM), University of Cape Town, Cape Town, South Africa

**Keywords:** Epidemiological reports, Outbreak, Infectious diseases, Arboviral disease, Viral haemorrhagic fever, Epidemic, Epi Alert

## Abstract

**Objectives:**

Timely, clinically relevant outbreak intelligence is critical in an evolving infectious disease landscape. Over twelve years, the Amsterdam UMC Centre of Tropical Medicine and Travel Medicine produced weekly *Epi Alerts* (EAs), summarizing reports for travel and tropical medicine clinicians. We analysed these EAs to describe pathogen and disease distributions, explore temporal and geographic trends and reporting biases, and evaluate cited source accessibility.

**Methods:**

We conducted a retrospective analysis of all EAs (April 2013-December 2024). Data from 454 bulletins yielded 10,619 entries classified by pathogen type, disease category, and location. The study was reported in accordance with the STROBE guidelines for observational studies.

**Results:**

Viral infections predominated (7,234/10,619; 68%), followed by bacterial (2,562; 24%), parasitic (646; 6%), and fungal (83; 1%) diseases. Arboviruses comprised 46% of viral entries (3,312/7,234), mainly dengue (1,210; 17%), chikungunya (328; 5%), and West Nile fever (314; 4%). Viral haemorrhagic fevers accounted for 17% (1,209/7,234) of all viral entries, predominantly Ebola virus disease (321; 4%), yellow fever (292; 4%), and Crimean–Congo haemorrhagic fever (246; 3%). Measles featured prominently (1,114/10,619; 11%). Reporting favoured English-language sources with higher internet visibility and well-resourced surveillance systems. Rare/emerging pathogens (e.g. Powassan, Oropouche, Kyasanur Forest disease viruses) were consistently captured. At analysis, 67% of hyperlinks were defunct (7,150/10,619).

**Conclusions:**

Although two-thirds of cited hyperlinks had become at the time of analysis, the EA archive offers durable, clinically relevant outbreak intelligence supporting diagnosis, travel advice, education. While selective, it captures emerging, rare and geographically unexpected infections relevant to clinical reasoning. Integration into ESCMID's Emerging Infections Subcommittee since mid-2025 ensures continuity and supports development into a searchable, continuously updated resource.

## Highlights

• 12-year outbreak database supports clinical reasoning for emerging infections.
• Viral infections predominate; arboviruses and VHFs drive alerts.
• Emerging pathogens consistently captured: e.g. Zika, Powassan, Oropouche virus.
• Two-thirds of source hyperlinks became defunct over time.
• Program integrated into ESCMID for continuity and standardisation.

## 1 Introduction

The epidemiology of infectious diseases is constantly shifting [1,2], driven by forces beyond host factors and pathogen evolution, including climate change [3], armed conflict [4] and international travel and migration [5]. The COVID-19 pandemic exemplified this dynamism, altering incidence patterns of other infectious diseases and disrupting routine prevention and treatment programmes [[6], [7], [8], [9]]. These shifts highlight the need for effective surveillance systems and rapid reporting to support timely clinical and public health responses [[10], [11], [12]].

Textbooks and journal reviews remain essential for background knowledge but are inherently static and rapidly outdated. Subscription platforms such as UpToDate [13] and GIDEON [14] provide more current summaries, yet often lag behind real-time epidemiological developments due to editorial and peer-review processes. Conversely, outbreak feeds including ProMED [15], BEACON [16] and OutbreakNewsToday [17] offer near-real-time reporting but may overwhelm readers with duplicate or locally confined events and increasingly limited accessibility due to paywalls. The central challenge therefore lies in curating outbreak intelligence that is both timely and clinically relevant.

To address this need, the Amsterdam UMC Centre of Tropical Medicine and Travel Medicine (‘Tropencentrum’; AUMC-TC) initiated weekly Epi Alerts (EAs), curated by senior infectious diseases physicians, to inform clinicians about outbreaks and epidemiological shifts relevant to travel medicine and potential disease presentation in returning travellers. The reports compile infectious disease updates from publicly available sources, including World Health Organization (WHO), European Center for Disease Prevention and Control (ECDC), Centers for Disease Control and Prevention (CDC), Program for Monitoring Emerging Diseases (ProMED) and OutbreakNewsToday (reporting up to January 2025).

EAs focus on new outbreaks or unexpected epidemiological changes and therefore usually exclude common endemic diseases unless marked by unusual features, such as large outbreaks or spread into previously non-endemic regions. Conversely, small outbreaks of non-endemic diseases (e.g. autochthonous chikungunya in Europe) are included. Consequently, EAs are not suited for general burden estimation and are not exhaustive, but provide a curated record of emerging infections and changes in endemicity over time. The EAs gained increased readership and are distributed via email to an internal and professional network and through social media [18]. Since mid-2025, they have been incorporated into the activities of the European Society for Clinical Microbiology and Infectious Diseases’ (ESCMID) Emerging Infections Subcommittee (EIS), with a team-based editorial process and wider distribution [19].

The weekly EAs provide timely, clinically relevant outbreak intelligence for practice in travel and tropical medicine. To enable retrospective interrogation across time and geography, we transformed the alerts into a searchable database. This study presents a retrospective descriptive analysis of EAs published between April 2013 and December 2024. The primary objective was to describe the distribution of reported diseases and pathogens within a dataset deliberately focused on outbreaks, novel pathogens and unusual epidemiological patterns rather than endemic disease burden. Secondary objectives were to explore temporal and geographic trends, identify reporting biases and underrepresented disease groups, and assess the long-term durability and accessibility of source material.

## 2 Methods

### 2.1 Study design

We conducted a retrospective observational study of publicly available EAs from April 2013 to December 2024, reported according to STROBE guidelines [20] (Supplementary File S1). Descriptive analyses were performed after data cleaning to summarise dataset characteristics and assess potential reporting bias.

### 2.2 Outcomes and definitions

The primary outcome was the distribution of reported diseases and pathogens. Secondary outcomes included temporal and geographic trends, identification of emerging or rare infections, and long-term accessibility of cited sources.

Pathogen types (viral, bacterial, parasitic, fungal, other) followed WHO and ICD-10/11 classifications. Disease categories were predefined: viral haemorrhagic fevers (VHF) followed the WHO list (including Ebola, Marburg, Lassa, Crimean–Congo haemorrhagic fever, yellow fever, Rift Valley fever, and related viruses); arboviral diseases were defined as infections caused by arthropod-borne viruses (e.g. Flaviviridae, Togaviridae); and vaccine-preventable diseases (VPDs) followed WHO definitions.

“Emerging, novel, or rarely reported” viral infections were defined as those outside common arboviral/VHF categories, with <100 entries over the study period, and either listed in the WHO R&D Blueprint or newly characterised or geographically reported. A full list of included diseases is provided in Supplementary Table S3.

### 2.3 Data sources

EAs were compiled from publicly available sources including WHO Disease Outbreak News, ECDC reports, CDC (HAN, MMWR), ProMED-mail, Outbreak News Today, national public health agencies (e.g. RIVM, RKI, UKHSA), and relevant peer-reviewed reports. Each EA entry contained a hyperlink to the primary source of the report. Hyperlinks pointed to the original source and not to supplementary information. In a small number of cases (<1%), the source was an email bulletin or internal communication without a public URL; these entries were retained with the source type recorded but no hyperlink. The ECDC Communicable Disease Threats Report was excluded during extraction to avoid duplication.

### 2.4 Data extraction and management

The unit of analysis was individual outbreak events within alerts. Extracted variables included disease/pathogen, pathogen type, location, date, case counts, source, and alert date. Only the initial source page was used for standardisation.

Diseases were categorised non–mutually exclusively. Geographic regions followed the UN M49 geoscheme; Russia and Turkey were classified as Europe, and Oceania included Australia, New Zealand, and Pacific Island states.

Data extraction from PDFs was manual, supported by a standardised ChatGPT-based prompt (Supplementary file S2), with all outputs verified against original reports [21]. When URLs were inaccessible, EA content was used.

### 2.5 Descriptive analysis

Descriptive statistics (means [SD], medians [IQR], proportions) were computed using Microsoft Excel [22] for Microsoft 365 (Microsoft Corporation, Redmond, WA, USA) and IBM SPSS Statistics [23] version 28.0 (IBM Corp., Armonk, NY, USA).

## 3 Results

A total of 454 EAs were published between April 2013 and December 2024, corresponding to 75% (454/609) of the weeks within the study period. A total of 10,619 disease entries yielded a median number of 23 entries per alert (IQR 19-27), with a median number of 41.5 EAs per year (IQR 34.5-43). Table 1, Table 2 show the categorised information sources and mentioned locations, respectively. At the time of analysis (January-April 2025), at least two-thirds (7,150/10,619) of all URLs were defunct. This was due to a combination of service changes (ProMED), domain migrations (OutbreakNewsToday) and link rot [24] in general.

**Table 1:** Sources cited in the Epi Alerts.

Source category	% of entries	Number of entries	Notes
Surveillance systems and reporting networks	37	3,892/10,619	ProMED as sole contributor
Private healthcare websites	26	2,791/10,619	OutbreakNewsToday accounted for 99% of entries
Official health organizations	23	2,440/10,619	
National and local media	10 (5 each)	1,072/10,619	More frequent in early years (2013–2014)
Academic institutions	3	370/10,619	
Governmental health agencies	0.4	40/10,619	
Non-governmental organizations	0.1	9/10,619	
Unknown/Untraceable	<0.1	5/10,619	No traceable source

**Table 2:** Geographic distribution of entries, 2013-2024.

Continent	Total entries (% of 10,619)	Top C/Ts (% of total entries per continent)	Median entries per C/T (IQR) over 12 years
Asia	2,817 (27)	India (17%; 485/2,817), Pakistan (10%; 269/2,817), Philippines (9%; 246/2,817)	21 (7–76)
North America	2,294 (22)	USA (68%; 1,563/2,294), Canada (7%; 170/2,294), Panama (5%; 115/2,294), Mexico (4%; 100/2,294), Dominican Republic (3%; 60/2,294)	2 (0-10)
Africa	2,123 (20)	DRC (16%; 332/2,123), Nigeria (13%; 270/2,123), South Africa (5%; 113/2,123)	17 (9–37)
Europe	1,455 (14)	United Kingdom (13%; 195/1,455), Spain (6%; 90/1,455), France (6%; 86/1,455), Russia (5%; 71/1,455)	7 (2–22)
South America	809 (8)	Brazil (37%; 299/809), Argentina (14%; 117/809), Venezuela (9%; 71/809), Colombia (7%; 59/809)	44 (7–59)
Oceania	559 (5)	Australia (52%; 289/559), New Zealand (14%; 80/559), Fiji (9%; 50/559)	5 (2–15)
Antarctica	0 (0)	Not available	Not available

C/T = countries/territories, USA = United States of America, DRC = Democratic Republic of the Congo, IQR = interquartile range.

Among the top ten most frequently reported diseases per pathogen type (Table 3), viral infections predominated, followed by bacterial, parasitic, and fungal diseases; fewer than 1% were classified as 'other,' including outbreaks due to toxins and prion diseases.

**Table 3:** Top ten diseases/pathogens per category, total entries: 10,619.

Pathogen Type (# of entries; % of total)	Disease	Number of entries	% of group total
Viral (7,234; 68)	Dengue	1,210	17
	Measles	1,114	15
	Chikungunya	328	5
	Ebola virus disease	321	4
	West Nile fever	314	4
	Hantavirus disease	301	4
	Yellow fever	292	4
	Rabies	288	4
	Zika	280	4
	MERS	255	4
Bacterial (2,562; 24)	Cholera	602	23
	Rickettsiosis	159	6
	Plague	124	5
	Diphtheria	124	5
	Leptospirosis	123	5
	Pertussis	122	5
	Typhoid fever	107	4
	Salmonellosis	98	4
	Anthrax	94	4
	Legionellosis	92	4
Fungal (83; <1)	Coccidioidomycosis	25	30
	Candidiasis (*Candida auris*)	13	16
	Histoplasmosis	12	14
	Blastomycosis	11	13
	Sporotrichosis	5	6
	Emergomycosis (*Emergomyces canadensis*)	3	4
	Mucormycosis	3	4
	Pulmonary aspergillosis	3	4
	Aspergillosis	2	2
	Fungal meningitis	2	2
Parasitic (646; 6)	Malaria	218	34
	Leishmaniasis	96	15
	Primary amoebic meningoencephalitis (*Naegleria fowleri*)	59	9
	Cyclosporiasis	44	7
	Trichinellosis	33	5
	Cryptosporidiosis	30	5
	Chagas' disease	26	4
	Rat lungworm disease (*Angiostrongylus cantonensis*)	23	4
	Schistosomiasis	20	3
	Human African trypanosomiasis	19	3

### 3.1 Viral diseases - arboviral diseases

Nearly half of all reports on viral diseases concerned arboviruses, 46% (3,312/7,234), predominantly dengue (37%; 1,210/3,312), chikungunya (10%; 328/3,312), and West Nile virus (WNV; 9%; 314/3,312). In total, 43 different arboviral pathogens were recorded, 13 only once, illustrating the breadth of reporting and the inclusion of rare or emerging agents. Over time, the five most-frequently reported arboviruses showed marked changes in frequency, including the characteristic rise and fall of Zika virus disease in 2016 and a sharp decline in dengue reports after initial peak periods.

Geographically, Europe accounted for 434 arboviral entries and the United States of America (USA) for 335, with WNV dominating in both (39% [168/434] and 35% [118/335], respectively). Latin America and the Caribbean contributed 700 entries, of which dengue, yellow fever, chikungunya and Zika comprised 91% (637/700).

### 3.2 Viral diseases - viral haemorrhagic fevers

**Fig. 1:**
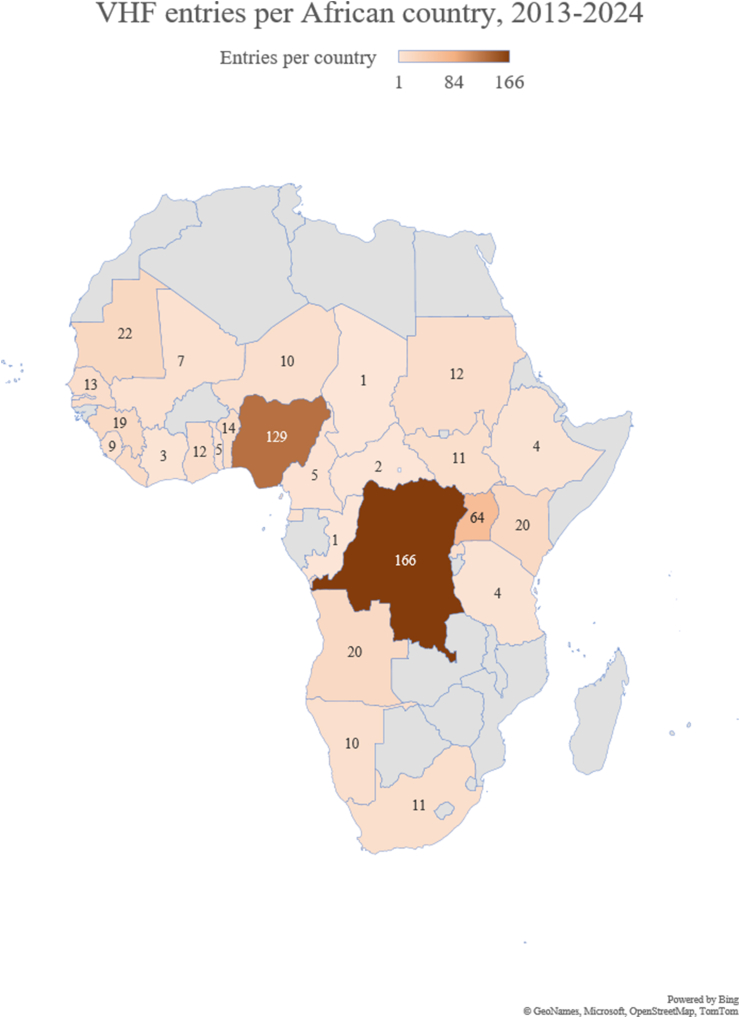
VHF entries per African country, 2013-2024.

**Fig. 2:**
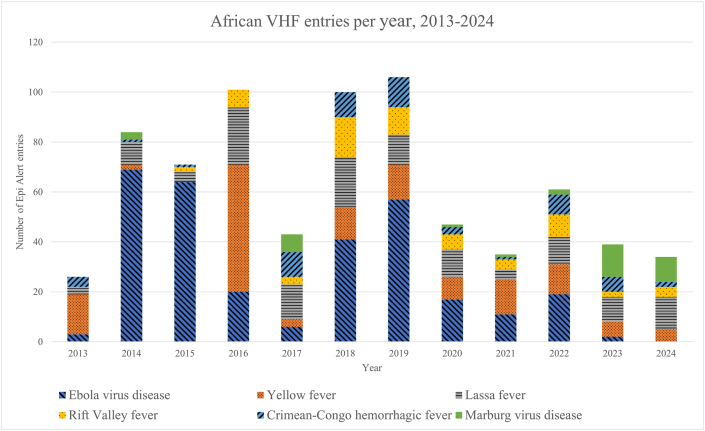
Annual African VHF entries.

**Fig. 3:**
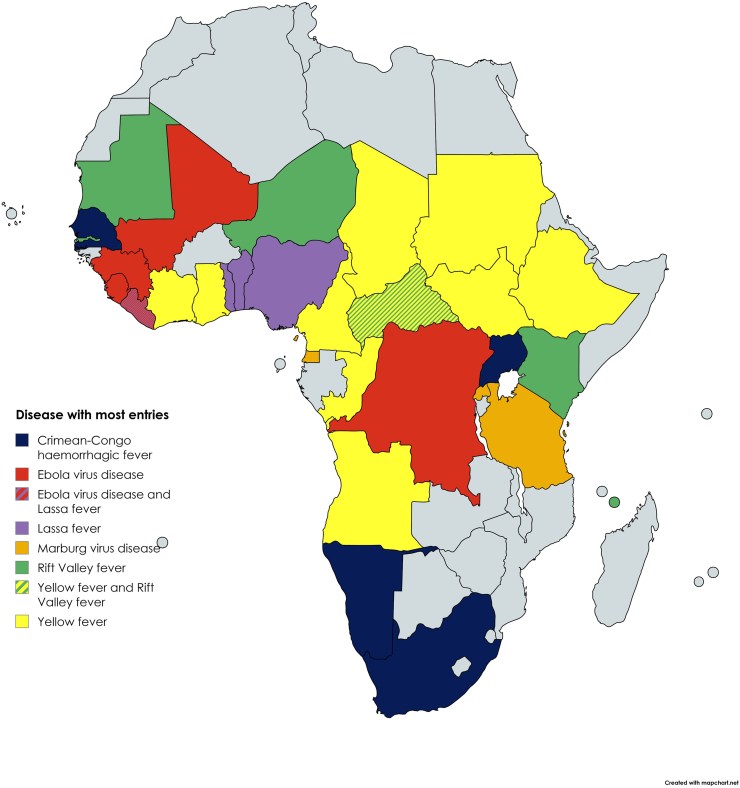
Most frequently reported VHF's per country.

VHFs comprised 17% (1,209/7,234) of all viral disease entries, of which 57% (691/1,209) were arboviral diseases (mostly yellow and Crimean-Congo haemorrhagic fever (CCHF). Within the VHF group, Ebola virus disease (27%; 321/1,209), yellow fever (24%; 292/1,209), CCHF (20%; 246/1,209) and Lassa virus disease (12%; 145/1,209) accounted for the majority of entries. Africa contributed nearly two-thirds (62%; 751/1,209) of these reports, followed by Asia (22%; 264/1,209) and South America (10%; 120/1,209); the most-frequently cited African countries are shown in Fig. 1. Weekly event reporting volume peaked between 2014 and 2019 and was lower after the pandemic. In Africa, Yellow fever peaked in 2016 and Ebola virus disease was the most frequently reported in six of twelve years (Fig. 2). The most-frequently reported VHFs per country are depicted in Fig. 3. Entries from Asia were dominated by CCHF (58%; 153/264), followed by Kyasanur Forest disease (22%; 59/264) and severe fever with thrombocytopenia syndrome (SFTS; 10%; 27/264).

### 3.3 Novel, emerging and rarely reported non-respiratory viruses

**Fig. 4:**
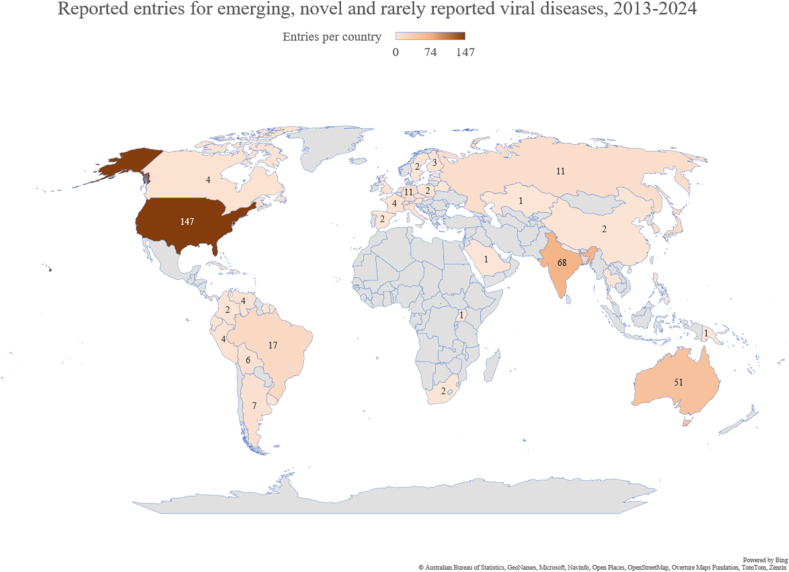
Number of mentionings of novel and rarely reported viral diseases, 2013-2024.

Emerging and rare viral diseases (Table 4) were reported 487 times. Ninety-seven percent (473/487) were traceable to a specific country (Fig. 4). The USA was the most-frequently mentioned country (31%; 147/473) and had the greatest diversity of pathogens (n = 13), predominantly neurotropic arboviruses led by Eastern equine encephalitis (33%; 49/147) and Powassan virus disease (20%; 30/147). India (14%; 68/473) and Australia (11%; 51/473) ranked second and third, with Kyasanur Forest disease (87%; 59/68) predominating in India and Ross River fever (57%; 29/51) in Australia. Africa was represented by three single reports from South Africa (Shuni and Middelburg virus disease) and Uganda (Ntwetwe virus disease).

**Table 4:** Emerging, novel and rarely reported viruses, 2013-2024.

Pathogen/disease	Number of entries (% of 487)
Kyasanur forest disease	59 (12)
Tick-borne encephalitis	58 (12)
Eastern equine encephalitis	54 (11)
Oropouche virus	38 (8)
Ross River virus	33 (7)
Nipah virus	31 (6)
Powassan virus encephalitis	30 (6)
Severe fever with thrombocytopenia syndrome	27 (6)
St. Louis encephalitis	22 (5)
Jamestown Canyon virus	17 (3)
Mayaro	13 (3)
Murray Valley encephalitis	13 (3)
La Crosse encephalitis	10 (2)
Heartland virus disease	8 (2)
Western equine encephalitis	8 (2)
Barmah Forest virus	6 (1)
Borna virus	6 (1)
Human rat hepatitis E	6 (1)
Bourbon virus	5 (1)
Usutu virus	5 (1)
Kunjin virus	4 (1)
Sindbis virus	3 (1)
Venezuelan equine encephalitis	3 (1)
Venezuelan haemorrhagic fever	3 (1)
Alkhurma virus	2 (<0.5)
Argentine haemorrhagic fever (Junin virus)	2 (<0.5)
Bolivian haemorrhagic fever (Machupo virus)	2 (<0.5)
Borealpox	2 (<0.5)
Madariaga	2 (<0.5)
Alongshan virus	1 (<0.5)
Chandipura virus	1 (<0.5)
Chapare haemorrhagic fever	1 (<0.5)
Colorado tick fever	1 (<0.5)
Echarate virus	1 (<0.5)
Everglades virus	1 (<0.5)
Iquitos virus	1 (<0.5)
Keystone virus	1 (<0.5)
Manych virus	1 (<0.5)
Middelburg virus	1 (<0.5)
Ntwetwe virus	1 (<0.5)
Shuni virus	1 (<0.5)
Toscana virus	1 (<0.5)
Wetland virus	1 (<0.5)
Yezo virus	1 (<0.5)

### 3.4 Arboviral encephalitides

**Fig. 5:**
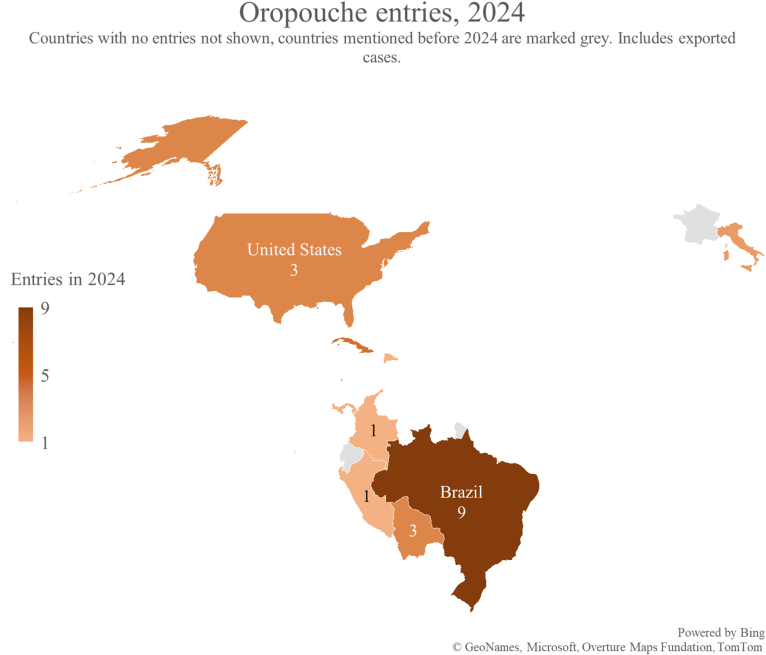
Number of mentionings of Oropouche virus in 2024.

A total of 747 reports concerned arboviral encephalitides. West Nile virus (WNV) accounted for 42% (314/747), followed by Japanese encephalitis (JE; 19%; 144/747) and tick-borne encephalitis (TBE; 8%; 58/747). Geographically, most reports originated from North America, Europe, and Asia. Of the WNV entries, 95% (297/314) came from North America (127/314; 40%) and Europe (170/314; 54%), with similar yearly rates. JE reports were mainly from Asia (85%; 123/144); in 2016, arboviral encephalitides entries from Asia briefly surpassed North America and Europe due to a marked rise in JE reports. TBE entries came almost exclusively from Europe (93%; 54/58). South America contributed few reports overall but showed a recent rise associated with the rise of Oropouche virus in 2024, which is shown in Fig. 5.

### 3.5 Vaccine-preventable diseases (VPDs)

**Fig. 6:**
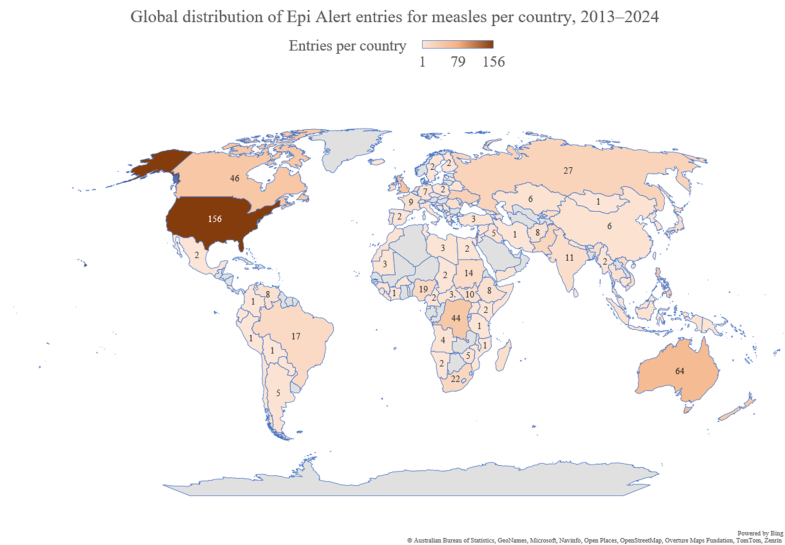
Number of mentionings of measles per country, 2013-2024.

VPDs made up 57% of all viral entries (4,109/7,234), which dropped to 36% (2,569/7,234) when excluding diseases for which vaccine indications were not (yet) routinely introduced, such as SARS-CoV-2, dengue, mpox, and respiratory syncytial virus. Among VPDs, measles was the most frequently reported disease (1,114/4,109; 27%), followed by yellow fever (292/4109; 7%) and rabies (288/4,109; 7%). Fig. 6 shows the number of measles entries per country. Entries came from every continent, highlighting global measles activity and areas of new introductions.

Bacterial VPDs (n = 441) were predominated by diphtheria (28%; 124/441), pertussis (28%; 122/441) and typhoid fever (25%; 107/441), with North America (26%; 114/441), Africa (24%; 105/441) and Asia (22%; 95/441) forming the top three.

## 4 Discussion

This study examined the value of a database containing information extracted from weekly EAs published between 2013 and 2024. The EA database offers practical benefits for clinical care in travel and tropical medicine. It supports early recognition of emerging or unusual infections by highlighting rare, novel, or geographically unexpected pathogens, from Powassan virus in North America to Oropouche virus in South America, helping clinicians consider differential diagnoses that might otherwise be missed in acutely ill returning travellers. It also strengthens travel and pre-travel advice by capturing dynamic shifts in disease distribution, including arbovirus introductions into Europe and vaccine-preventable disease surges in traditionally low-incidence regions. Finally, it supports clinical teaching by providing a curated, durable record of outbreak intelligence spanning common arboviruses to rare encephalitides, helping translate epidemiological signals into real-world diagnostic challenges.

As expected, given the purpose-driven design of the EAs, infectious diseases with the highest global health burdens, such as malaria, tuberculosis, and HIV/AIDS, were underrepresented in the database, despite each accounting for approximately 50 million disability-adjusted life years globally [25]. None ranked among the ten most frequently reported diseases, in contrast to dengue, measles, and cholera. This reflects the EA's explicit focus on outbreaks, geographic spread beyond endemic zones, and unexpected epidemiological changes rather than on chronic or stable endemic disease burdens.

This purpose-driven focus was also reflected in an over-representation of VHFs, which are rare compared with arboviral diseases such as dengue and chikungunya that affect millions annually [26,27]. Nevertheless, the ratio of arboviral diseases to VHFs in the dataset when excluding overlapping entries was 5:1, consistent with the disproportionate attention VHFs receive because of their high case fatality rates and epidemic potential. Diseases simultaneously classified as both arboviral and and VHF were counted only once, in the VHF group, for the purpose of that specific comparison. This pattern aligns with the EA's original intent to prioritise unusual, high-impact outbreak signals over background epidemiology of endemic diseases. Consequently, the database is not suited for estimating disease incidence but is well suited for retrospective analyses of which pathogens caused outbreaks or were introduced into new geographic areas over time. This is illustrated by the representation of rare and emerging pathogens reported worldwide, albeit with a disproportionate contribution from countries with stronger surveillance systems, higher internet visibility or English language reporting, e.g. the USA, India, and Australia. The EA database includes numerous arboviral encephalitides (e.g. Powassan and Jamestown Canyon virus disease) as well as emerging or novel viruses such as Wetland and Yezo virus in Northeast Asia and Oropouche virus in Latin America, supporting its role as an early-warning and educational resource for clinicians interested in emerging or rare infections.

With almost one-third of all entries, VPDs were well represented. Measles, by far the most frequently reported VPD, was shown to be introduced in many previously non-endemic regions. A clear reporting bias was evident: D.R. Congo, with the highest global measles incidence, had 1,344,162 cases between 2013 and 2024, compared to 3,032 in the USA, a ratio of 443:1 [28]. Yet, the USA was more frequently mentioned due to the steep increase in cases following the decline in vaccination rate [29], whereas D.R. Congo cases were reported only when unusually large outbreaks occurred.

Parasitic and fungal diseases were infrequently reported, which was even more apparent when malaria was excluded. This was anticipated, as these pathogen types account for a smaller share of global disease burden and rarely cause epidemics. Most entries related to these groups were included due to their clinical novelty.

An important observation concerns the durability of online sources. At least two-thirds of cited hyperlinks were nonfunctional at the time of data extraction, reflecting the well-documented problem of ‘link rot’ [24]. Though this limited retrieval of additional outbreak metrics and contextual details beyond what was preserved in the alerts, it underscores that real-time outbreak feeds do not constitute a durable record. Key variables (disease, location, source type and date) remained available within the EAs, highlighting that curated, self-contained databases retain their clinical and educational utility long after the original sources have disappeared. A searchable, publicly available EA database could support rapid clinical reasoning by establishing whether a disease has been reported in a given place and time period (numerator information). Incidence or prevalence estimates (denominator information) would be needed to quantify risk, but are not required for the initial question of recent presence, whether a large epidemic in Brazil or a small autochthonous cluster in France, both represented as single data points in this archive. While this approach lacks epidemiological granularity, a ‘yes/no’ signal can still be clinically useful for differential diagnosis in returning travellers; questions of scale can then be addressed with complementary sources, particularly for pathogens with high epidemic potential.

### 4.1 Limitations

The primary limitations are selection bias, the aforementioned reporting bias, and missing data due to defunct weblinks. The selection bias was partly intentional, as discussed, but also inherent in a then single-author model (now succeeded by an AI-supported editorial team approach) where the individual responsible for curation also served as editor and publisher, introducing subjective decision-making. This was reflected in several key findings: a nearly 50% decrease in reporting during the COVID-19 pandemic years, the skewed ratio of arboviral diseases to VHFs, the underrepresentation of high-burden diseases such as malaria, tuberculosis, and HIV/AIDS, geographic asymmetries in country-level data, and the underrepresentation of parasitic and fungal infections. These patterns were intentional and aligned with the primary goal of the EA, but also reflect personal choices made by the author/editor. The 25% gap in coverage was also a result of the single-author model, stemming from (personal) holidays, or clinical obligations. Relevant findings were usually covered in the following issue.

### 4.2 Future perspectives

Real-time incorporation of EAs would expand the database in both volume and utility, reduce workload, and mitigate data loss due to link rot. Building on the current searchable archive, the EA database has been implemented as a vector database, enabling flexible retrieval beyond standard spreadsheet queries and supporting rapid interrogation using Python, for example to determine whether a given pathogen has been reported in a specific country over a defined period. This searchable archive also supports retrieval-augmented approaches, in which new outbreak signals can be automatically compared with the historical database and ranked by clinical relevance.

Following the integration of the EA within the Emerging Infections Subcommittee (EIS) of ESCMID in July 2025, a study group under the supervision of the original author standardized EA creation and ensured continuity of publication beyond a single contributor's availability. The long-term objective is to develop an open-access, searchable, and regularly updated database for the ESCMID community. A relevant reference model is the Liverpool HIV Drug Interactions Checker [30], a globally recognized clinical decision-support tool; a comparable framework is currently being further developed for the EA database.

## CRediT authorship contribution statement

**Jelmer van Os:** Writing – original draft, Visualization, Software, Methodology, Investigation, Formal analysis, Data curation, Conceptualization. **Hanna K. de Jong:** Writing – review & editing, Supervision, Methodology, Formal analysis, Conceptualization. **Galadriel Pellejero-Sagastizabal:** Writing – review & editing. **José Ramón Paño-Pardo:** Writing – review & editing. **F-Xavier Lescure:** Writing – review & editing. **Thomas Hanscheid:** Writing – review & editing, Software, Data curation. **Martin P. Grobusch:** Writing – review & editing, Supervision, Methodology, Conceptualization. **Abraham Goorhuis:** Writing – review & editing, Supervision, Methodology, Formal analysis, Conceptualization.

## Ethical approval

Not required as no patient data was used for this work.

## Data statement

Data are available from the corresponding author upon reasonable request.

## Declaration of generative AI and AI-assisted technologies in the writing process

We have used AI (OpenAI's GPT, versions GPT-4.5, 5, 5.1 and 5.2), to enhance the language and readability of our manuscript. This application of AI technology did not replace any essential tasks such as producing scientific insights, analysing and interpreting data, or drawing scientific conclusions. The authors reviewed all AI-generated suggestions and take full responsibility for the final content. We disclose this writing assistance in accordance with journal guidelines.

## Funding source

None.

## Declaration of competing interest

The authors declare that they have no known competing financial interests or personal relationships that could have appeared to influence the work reported in this paper.
