# Supplementary material for: Analysing outbreak signals, 2013–2024: The amsterdam UMC centre for Tropical Medicine and Travel Medicine Epi Alert programme – an observational study
Source: New Microbes New Infect. 2026 May 4;71:101756. doi: 10.1016/j.nmni.2026.101756 (PMC13158387; doi:10.1016/j.nmni.2026.101756)
Supplement: Multimedia component 2 [file mmc2.docx]

**S2. AI-assisted extraction**

Epi Alert documents were processed between January and March 2025 using ChatGPT, an OpenAI large language model based on the GPT-4 family (specifically GPT-4 Turbo and its successor variant, GPT-4o), which was the active model used by ChatGPT during that period. The model was used to extract and structure epidemiological information (disease, location, and source) from PDF files according to predefined rules, including separate entries per location and standardized disease naming. The final prompt (after multiple revisions)

used was as follows:

Extract epidemiological information from the provided Epi Alert PDF and convert it into a structured table with the following specifications:

- *Columns: Disease and Location only (no additional columns).*
- *Each location must be listed as a separate row (no grouping of multiple locations in one entry).*
- *Locations must be formatted with the most specific administrative level first (city, state or province), followed by the country as the final element.*
- *In the Disease column, include only the disease name (no descriptors such as “fatal case,” “outbreak,” or “increase”).*
- *Standardize disease names as follows:*
  - *“Ebola” → “Ebola virus disease”*
  - *“Zika virus” → “Zika”*
  - *“Hantavirus” → “Hanta virus”*
- *If multiple reports refer to the same disease in different locations, list each location separately.*
- *If no specific location is provided (e.g., WHO or ECDC updates), leave the location field blank.*
- *Ensure consistent formatting across all entries.*
- *Do not infer missing data. Do not merge rows. Output only the table.*

All AI-assisted outputs were manually verified against source documents by a human extractor; identified discrepancies were corrected during verification. There were no explicit prompts used for data analysis, as this was done manually by the first author.
