# Supplementary material for: Analysing outbreak signals, 2013–2024: The amsterdam UMC centre for Tropical Medicine and Travel Medicine Epi Alert programme – an observational study
Source: New Microbes New Infect. 2026 May 4;71:101756. doi: 10.1016/j.nmni.2026.101756 (PMC13158387; doi:10.1016/j.nmni.2026.101756)
Supplement: Multimedia component 3 [file mmc3.docx]

**S3. Alphabetical list of all included diseases**

| Acinetobacter baumannii |
| --- |
| Acute encephalitis syndrome |
| Acute flaccid myelitis |
| Acute flaccid paralysis |
| Acute neurologic illness |
| Acute Respiratory Syndrome |
| Adenovirus |
| Aeromonas skin infections |
| African tick bite fever |
| Alkhurma virus |
| Alongshan virus |
| Amoebiasis |
| Anaplasmosis |
| Angiostrongylus cantonensis |
| Anisakiasis |
| Anncaliia algerae microsporidial myositis |
| Anthrax |
| Antibiotic resistance |
| Argentine hemorrhagic fever (Junin virus) |
| Aspergillosis |
| Australian bat Lyssavirus |
| Australian bat lyssavirus |
| Avian influenza |
| Avian influenza A (H10N8) |
| Avian influenza A (H5) |
| Avian influenza A (H5N1) |
| Avian influenza A (H5N2) |
| Avian influenza A (H5N6) |
| Avian influenza A (H6N6) |
| Avian influenza A (H7N9) |
| Avian influenza A (H9N2) |
| Babesiosis |
| Barmah Forest virus |
| Bartonella bacilliformis |
| Bartonella quintana |
| Bartonellosis |
| Baylisascaris procyonis |
| Blastomycosis |
| Bolivian hemorrhagic fever (Machupo virus) |
| Bordetella parapertussis |
| Borealpox |
| Borna virus |
| Borrelia miyamotoi |
| Botulism |
| Bourbon virus |
| Bovine tuberculosis |
| Brucellosis |
| Buruli ulcer |
| Campylobacteriosis |
| Candida auris |
| Carbapenem-resistant enterobacterales |
| Cat scratch disease (Bartonella henselae) |
| Chagas disease |
| Chandipura virus |
| Chapare hemorrhagic fever |
| Chikungunya |
| Chlamydia |
| Cholera |
| Ciguatera |
| Circovirus |
| Circovirus hepatitis |
| Clostridial myonecrosis |
| Clostridium difficile |
| Coccidioidomycosis |
| Cochliomyia hominivorax |
| Colorado tick fever |
| Counterfeit rabies vaccine |
| Creutzfeldt-Jakob disease |
| Crimean-Congo hemorrhagic fever |
| Crithidiasis |
| Cryptococcus tetragatii meningitis |
| Cryptosporidiosis |
| Cutaneous leishmaniasis |
| cVDPV |
| cVDPV1 |
| cVDPV2 |
| cVDPV3 |
| Cyclosporiasis |
| Cyclovirus |
| Cysticercosis |
| Dengue |
| Diarrhea |
| Diphtheria |
| Diphyllobothriasis |
| Dirofilariasis |
| Disseminated gonococcal disease |
| Dracunculiasis |
| Drug-resistant infections |
| E. coli |
| E. coli (EHEC) |
| Eastern equine encephalitis |
| Ebola virus disease |
| Echarate virus |
| Echinococcosis |
| Ehrlichia infections |
| Elephantiasis |
| Elisabethkingia anophelis |
| Emergomyces canadensis |
| Enterobacteriaceae |
| Enterovirus |
| Enterovirus A71 |
| Enterovirus D68 |
| Enterovirus, Echovirus 11 |
| Enterovirus, Echovirus 30 |
| Everglades virus |
| Fascioliasis |
| Filariasis (Mansonella ozzardi) |
| Flea-borne diseases |
| Food poisoning |
| Fungal meningitis |
| Gastroenteritis |
| Giant hornet stings |
| Gnathostomiasis |
| Gonococcal infection |
| Gonorrhea |
| Group A streptococcal infections |
| Haemophilus influenzae type B |
| Haff disease |
| Halicephalobus gingivalis |
| Hand, foot, and mouth disease (HFMD) |
| Hanta virus disease |
| Heartland virus disease |
| Hemolytic uremic syndrome |
| Hepatitis |
| Hepatitis A |
| Hepatitis B |
| Hepatitis C |
| Hepatitis E |
| Hepatitis of unknown origin |
| Herpes |
| Herpes B |
| Histoplasmosis |
| HIV |
| HIV drug resistance |
| HIV/AIDS |
| Hookworm |
| HTLV-1 |
| Human African trypanosomiasis |
| Human metapneumovirus |
| Human rat hepatitis E |
| Influenza |
| Influenza A (H1N1) |
| Influenza A (H1N2) |
| Influenza A (H3N2) |
| Invasive group A Streptococcus infection |
| Iquitos virus |
| Israeli spotted fever (Rickettsia conorii complex) |
| Jamestown Canyon virus |
| Japanese encephalitis |
| Japanese spotted fever (Rickettsia japonica) |
| Keystone virus |
| Klebsiella pneumoniae |
| Kunjin virus |
| Kyasanur forest disease |
| La Crosse encephalitis |
| Lassa fever |
| Legionellosis |
| Leishmaniasis |
| Leprosy |
| Leptospirosis |
| Listeriosis |
| Louse-borne relapsing fever |
| Lyme disease |
| Lymphatic filariasis |
| Lymphocytic choriomeningitis |
| Lymphogranuloma venereum |
| Mad cow disease (BSE) |
| Madariaga |
| Malaria |
| Manych virus |
| Marburg virus disease |
| Mayaro |
| Measles |
| Melioidosis |
| Meningitis |
| Meningococcal disease |
| Meningococcal disease (serogroup A) |
| Meningococcal disease (serogroup C) |
| Meningococcal disease (serogroup W) |
| Meningococcal disease (serogroup Y) |
| Meningococcal meningitis |
| Meningococcal meningitis (serogroup B) |
| Meningococcal meningitis (serogroup C) |
| Meningococcal meningitis (serogroup W) |
| Meningococcal meningitis (serogroup Y) |
| MERS-CoV |
| Middelburg virus |
| Mosquito-borne diseases |
| Mpox |
| Mpox clade I |
| Mpox clade I and II |
| Mpox clade Ib |
| MRSA |
| Mucormycosis |
| Mumps |
| Murine typhus (Rickettsia typhi) |
| Murray Valley encephalitis |
| Mycobacteria |
| Mycobacterium abscessus |
| Mycobacterium chimerae |
| Mycobacterium marinum |
| Mycoplasma pneumoniae |
| Myiasis |
| NDM-carrying Enterobacteriaceae |
| Necrotizing fasciitis |
| Neoehrlichosis |
| Nipah virus |
| Nodding disease |
| Norovirus |
| Novel coronavirus |
| Ntwetwe virus |
| Onchocerciasis |
| Ophidascaris robertsi |
| Opistorchiasis |
| Orientia infection |
| Oropouche |
| Paracoccidioidomycosis |
| Paralytic shellfish poisoning |
| Paratyphoid fever |
| Parechovirus |
| Parvovirus B19 |
| Pasteurella multocida |
| Pertussis |
| Plague |
| Pneumococcal disease |
| Pneumococcal meningitis |
| Pneumonia of unknown cause |
| Poliomyelitis |
| Powassan virus encephalitis |
| Primary amoebic encephalitis (Balamuthia mandrillaris) |
| Primary amoebic meningo-encephalitis (Naegleria fowleri) |
| Pseudomonas aeruginosa, extensively drug-resistant |
| Psittacosis |
| Puffer fish poisoning |
| Pulmonary aspergillosis |
| Q-fever |
| Rabies |
| Rat bite fever |
| Respiratory infections |
| Rickettsia parkeri |
| Rickettsiosis |
| Rift Valley fever |
| Rocky Mountain spotted fever (Rickettsia rickettsii) |
| Ross River virus |
| Rotavirus |
| RS-virus |
| Rubella |
| Salmonellosis |
| Sarcocystosis |
| SARS-CoV-2 |
| Scabies |
| Schistosomiasis |
| Scrub typhus |
| Severe fever with thrombocytopenia syndrome (SFTS) |
| Shigellosis |
| Shuni virus |
| Sindbis virus |
| Sparganosis |
| Sporotrichosis |
| Spotted fever (Rickettsia aeschlimannii) |
| Spotted fever (Rickettsia philippii) |
| St. Louis encephalitis |
| STD's |
| Streptococcus suis |
| Strongyloidiasis |
| Swine influenza A (H1N1) |
| Swine influenza A (H3N2) |
| Syphilis |
| Tetanus |
| Thelaziasis |
| Tick-borne encephalitis |
| Tick-borne pathogens |
| Tick-borne relapsing fever |
| Toscana virus |
| Toxic spider bites |
| Toxoplasmosis |
| Trichinellosis |
| Trichophyton mentagrophytes genotype VII (TMVII) |
| Tuberculosis |
| Tularemia |
| Typhoid fever |
| Undiagnosed hemorrhagic fever |
| Unknown disease |
| Usutu virus |
| Vector-borne diseases |
| Venezuelan equine encephalitis |
| Venezuelan hemorrhagic fever |
| Vibrio parahaemolyticus |
| Vibrio vulnificus |
| Viral conjunctivitis |
| Visceral leishmaniasis |
| West Nile virus |
| Western equine encephalitis |
| Wetland virus |
| WPV |
| WPV1 |
| Yellow fever |
| Yellow fever associated viscerotropic disease |
| Yersiniosis |
| Yezo virus |
| Zika |

Figure 1
